# Comparison of two frailty indices in predicting life-threatening morbidity and mortality among older patients undergoing elective high-risk abdominal surgery

**DOI:** 10.3389/fpubh.2023.1055001

**Published:** 2023-04-05

**Authors:** Chun-Qing Li, Hao Kong, Zhen-Zhen Xu, Jia-Hui Ma, Xue-Ying Li

**Affiliations:** ^1^Department of Anaesthesiology, Peking University First Hospital, Beijing, China; ^2^Department of Biostatistics, Peking University First Hospital, Beijing, China

**Keywords:** frailty indices, high-risk surgery, older patient, morbidity, mortality

## Abstract

**Background:**

Frailty predicts an increased risk of postoperative morbidity and mortality. Comparison of the predictive performance between two deficit accumulation models of frailty, the modified frailty index (mFI) and the revised-Risk Analysis Index (RAI-rev), is poorly understood. This study compared the predictive abilities of the above two frailty indices in predicting life-threatening morbidity and mortality among older patients following elective high-risk abdominal surgery.

**Methods:**

This retrospective cohort study extracted perioperative data of older patients (age ≥65 years) undergoing elective high-risk abdominal surgery at a single institution between January 2018 and December 2020. Preoperative frailty was screened by mFI and RAI-rev scoring systems. The primary outcome was the composite of postoperative life-threatening morbidity and mortality during hospitalization. Multivariable logistic regression analyses were performed to investigate the association of the two frailty indices with the primary outcome. Receiver-operating characteristic (ROC) curve was employed to test the predictive performances of the two frailty instruments in predicting the composite primary outcome. The difference between the area under the curves (AUCs) was assessed by DeLong’s test.

**Results:**

1,132 older patients (mean age, 73.4 ± 6.2 years; 63.9% male) were included. Of these, 107 (9.5%) developed postoperative life-threatening morbidity and mortality. In multivariable logistic regression analyses, rising continuous frailty scores (mFI: adjusted OR 1.319 per 0.09-point increase in score, 95% CI 1.151–1.511, *p* < 0.001; RAI-rev: adjusted OR 1.052 per 1-point increase in score, 95% CI 1.018–1.087, *p* = 0.002) as well as dichotomized frailty measures (mFI ≥0.27: adjusted OR 2.059, 95% CI 1.328–3.193, *p* = 0.001; RAI-rev ≥45: adjusted OR 1.862, 95% CI 1.188–2.919, *p* = 0.007) were associated with increased odds of the primary outcome separately. ROC curve analysis showed that the discrimination of mFI and RAI-rev scores for the life-threatening morbidity and mortality was poor and comparable (AUC: 0.598 [95% CI 0.569–0.627] vs. 0.613 [95% CI 0.583–0.641]; DeLong’s test: Z = 0.375, *p* = 0.7075).

**Conclusion:**

High mFI and RAI-rev scores were associated with an increased risk of life-threatening morbidity and mortality in older patients undergoing elective high-risk abdominal surgery. However, both frailty indices displayed poor discrimination for postoperative life-threatening morbidity and mortality.

## Introduction

With the rapid expansion of the aging population, frailty has constituted a critical public health issue for healthcare providers worldwide. Frailty is a multidimensional geriatric syndrome characterized by reduced physiologic reserve, accumulated deficits, and decreased resistance to stressors (1, 2). With older frail individuals increasingly presenting for surgical interventions, clinicians have to face the burden and challenges brought by frailty in perioperative settings (3, 4). Indeed, accumulating evidence demonstrates preoperative frailty is associated with increased risks of adverse system-centered outcomes (postoperative morbidity and mortality, prolonged hospital stay, readmissions, etc.) and patient-centered outcomes (disability, lower quality of life, etc.) across various surgical specialties (5–11). Preoperative frailty screening and interventions are strongly recommended across a wide range of surgical procedures, including elective high-risk procedures (12).

Compared with low-risk surgery, high-risk surgery exerts greater physiologic stress on older individuals and is prone to higher odds of major morbidity and mortality (13). It is imperative for clinicians and patients to adequately balance the risks and benefits of high-risk surgery during the shared decision-making process. Frailty assessment and its application in predicting postoperative outcomes have significant influences on the consideration of the tradeoff between the risks and benefits of surgery and the determination of overall goals of care for a patient, especially in the context of high-risk surgery. Furthermore, frailty screening can help guide the efficient allocation of perioperative care resources to high-risk patients as well as identify the modifiable domains as targets for tailored intervention to improve outcomes (12). A careful selection of a practical frailty screening tool can help improve the safety and quality of high-risk surgery among the vulnerable older population.

Over the past decades, dozens of frailty assessment tools have been developed (5, 6, 14–21). Generally, almost all frailty measurements are based on the subsections of the two most accepted frailty models, i.e., the frailty phenotype (20) and the frailty index (21). The frailty phenotype defines frailty as a pre-disability syndrome, which is suitable for the initial screening of non-disabled individuals at risk of adverse events (20); however, the presence of disability conditions may weaken its predictive ability for poor outcomes due to a sort of “ceiling effect” (22). The frailty index identifies frailty by evaluating “accumulated deficits” across multiple dimensions such as functional, medical, cognitive, nutritional, and social domains (21). Both the modified frailty index (mFI) and revised-Risk Analysis Index (RAI-rev) scoring systems are based on the deficit accumulation model of frailty. As a shortened scale derived from the Canadian Study of Health and Aging Frailty Index, the mFI comprises 11 components: 10 comorbidities and 1 item on functional status (5). RAI-rev is derived from the original RAI and consists of multiple domains, including aging, comorbidities, nutrition, cognitive ability, and functional and social status (6, 14). Evidence demonstrates that both frailty indices can predict adverse postoperative outcomes (5–10). Additionally, the two frailty indices can be readily obtained from routine clinical practice, either prospectively or retrospectively (5, 6). Given the association of the two frailty indices with poor postsurgical outcomes and the feasibility of their implementation, it is expected that they have the potential to efficiently utilize existing resources and improve the safety and quality of high-risk surgery in older patients. In highly-efficient perioperative settings, it is unrealistic and unnecessary to apply both frailty indices to a particular patient. Thus, it will be interesting to explore which one is more suitable to use in the context of high-risk operations. As far as we know, there is a lack of evidence on the head-to-head comparison between the above two frailty indices in predicting serious morbidity and mortality among older patients undergoing high-risk surgery.

The present study aimed to compare the performances of mFI and RAI-rev in predicting the composite outcome of life-threatening morbidity and mortality in older patients who underwent elective high-risk abdominal surgery.

## Methods

This retrospective cohort study was conducted in Peking University First Hospital, a tertiary general hospital in Beijing, China. The ethical approval was provided by the Biomedical Research Ethics Committee of Peking University First Hospital (2019 [296]). The Ethics Committee agreed to waive the written informed consent from the participants due to the retrospective nature of the study and that no patient follow-up was carried out. The privacy of participants was strictly observed.

### Patient selection

Older patients (≥65 years of age) who received elective high-risk abdominal surgery (including general and urologic surgical procedures) in Peking University First Hospital from January 2018 to December 2020 were screened for study inclusion. We utilized the Operative Stress Score (OSS) system to select patients who underwent high-risk surgery. OSS system rates common operations according to physiologic stress, i.e., OSS1, very low stress; OSS 2, low stress; OSS 3, moderate stress; OSS 4, high stress; and OSS 5, very high stress (23). We defined high-risk surgery as those with high or very high stress, i.e., OSS 4 and 5 operations (Supplementary Table S1). Patients with missing or incomplete important data were excluded. All data were extracted from our electronic medical records.

### Frailty measurement by modified frailty index

The 11 frailty deficits contained in the mFI were collected based on the National Surgical Quality Improvement Program definitions; each component was allocated the same weight of 1 point (Supplementary Table S2). The mFI score was calculated by dividing the sum of deficits present by 11. The resulting index thus ranges from 0 to 1.0, with higher scores indicating increasing frailty (5). Additionally, we dichotomized the continuous mFI scores into two categories based on our previous work (24), i.e., non-frail (mFI score <0.27) and frail (mFI score ≥0.27).

### Frailty measurement by revised-Risk Analysis Index

RAI-rev score was obtained by evaluating 11 variables, i.e., age, sex, cancer, unintentional weight loss, poor appetite, renal failure, congestive heart failure, shortness of breath, residence other than independent living, functional status, and cognitive decline. The weight of each item is detailed in Supplementary Table S3. The total score is between 0 and 81, with higher scores implying a more severe frailty condition (6). In the event that more than one operation was performed on a patient during the same hospitalization period, only the first round of the surgery and the corresponding preoperative RAI-rev score were measured. We dichotomized the continuous RAI-rev scores into non-frail (RAI-rev score <45) and frail (RAI-rev score ≥45) according to previous literature (7).

### Covariates

Baseline characteristics not covered by mFI or the RAI-rev were collected, including ASA physical status classification, body mass index (BMI), smoking status, current alcoholism, other major comorbidities, and main laboratory test results. Intraoperative data were also gathered, including risk stratification of surgery categorized by OSS (i.e., OSS 4 and 5) (23), duration of surgery, type of anesthesia, estimated blood loss, and intraoperative blood transfusion.

### Postoperative outcomes

The primary endpoint was the composite postoperative outcome of life-threatening morbidity and mortality during hospitalization, i.e., defined as Clavien-Dindo (CD) grade IV and V complications (25). CD grade IV complications refer to life-threatening morbidity requiring intermediate care/intensive care unit (ICU) management, consisting of single and multiple organ dysfunction. CD grade V complication means the death of a patient (25). For patients who experienced multiple morbidities, we included the most serious one in the analysis. The clinical diagnostic criteria for life-threatening morbidity are detailed in Supplementary Table S4. The secondary outcomes included time to life-threatening morbidity and mortality (i.e., the time interval from surgery to the occurrence of life-threatening morbidity and mortality), postoperative ICU admission, prolonged hospital stay (defined as greater than the 75th percentile of the length of hospital stay for each type of surgery), and adverse discharge destination (defined as discharge to destinations other than home, such as skilled care facility or other hospitals).

### Statistical analysis

The baseline and perioperative variables were compared between patients with life-threatening morbidity and mortality and those without. We also compared the postoperative outcomes between frail and non-frail patients according to the dichotomized frailty measures. Continuous variables were analyzed with the independent samples t-test or Mann–Whitney U test; the Kolmogorov–Smirnov test was performed to check for normality. Categorical variables were analyzed using *χ*^2^ tests, continuity-corrected *χ*^2^ tests, or Fisher’s exact tests. Time-to-event variables were analyzed using the Kaplan–Meier estimator, with differences between groups assessed by the log-rank test.

Due to the non-normal distribution of the data, we used Spearman’s correlation analysis to test the correlation of the two continuous frailty measures. The agreement of dichotomized measures was evaluated using the percentage of agreement and Cohen’s Kappa coefficient.

We used univariate and multivariable logistic regression analyses to calculate odds ratios (ORs) and 95% confidence intervals (CIs) of frailty in predicting life-threatening morbidity and mortality. We first analyzed the mFI score as a continuous variable and calculated the ORs and 95% CIs for the primary outcome per one-unit increase in mFI scores. Herein, to facilitate the clinical application of our findings, we defined one unit of the mFI score as 0.09 points (i.e., corresponding to 1 frailty trait). Potential confounding factors (not including the 11 variables covered by mFI) were screened by univariate analyses and tested for multicollinearity by variance inflation factor analysis. Factors with *p* values <0.10 in univariate analyses were then included in a multivariable model to examine the covariate-adjusted relationship between the rising mFI score and the primary outcome. Next, we analyzed the mFI score as a dichotomized measure and built another multivariable model to determine the adjusted association of frailty with the primary outcome. Likewise, the above statistical method was employed to explore the relationship of RAI-rev scores with the primary outcome. Herein, we defined each unit of the RAI-rev score as 1 point; similarly, the 11 variables included in RAI-rev were not enrolled in the corresponding multivariable models. All the multivariable analyses were performed with the backward stepwise method.

Besides, we conducted survival analysis to further explore the time effect of frailty (i.e., the two dichotomized frailty measures) on postoperative life-threatening morbidity and mortality. Herein, we adopted the 30-day life-threatening morbidity and mortality after surgery as the primary outcome in the survival analysis since almost all the primary endpoint events occurred within 30 days postoperatively in our study. The time to the endpoint event was calculated from the time of surgery to the date of the occurrence of life-threatening morbidity and mortality. Patients who were not observed to experience the primary outcome within 30 days and remained hospitalized after 30 days as well as those who did not undergo the endpoint event during hospitalization and were discharged from hospital within 30 days were all censored accordingly. Univariate analyses were performed using the Kaplan–Meier estimator with comparisons between frail and non-frail patients assessed by log-rank test. After multicollinearity screening, potential confounding factors (set at *p* < 0.10 in log-rank tests) were included in multivariable Cox proportional hazards regression models to examine the adjusted relationship of frailty with the primary outcome. The factors included in the mFI and RAI-rev were not entered into the corresponding multivariable Cox regression model.

The predictive performances of mFI and RAI-rev were tested using the receiver-operating characteristic (ROC) curve analysis. The area under the ROC curve (AUC) was measured to test the discriminative power (ability to classify correctly) for the primary outcome. An AUC value of 1 indicates the best discrimination, whereas a value of 0.5 indicates that the predictor is no more reliable than chance. Generally, a predictor may be considered useful in clinical decision-making when the AUC exceeds 0.7 (26). Differences between AUCs were assessed by the DeLong’ test.

For all analyses, two-tailed *p* values <0.05 were considered statistically significant. *p*-values were not corrected since no multiple comparison test was involved. All statistical analyses were performed with the SPSS version 26.0 (IBM Corp., Armonk, NY, United States) and MedCalc version 19.05 (Ostend, Belgium).

## Results

### Patient characteristics

**Figure 1:**
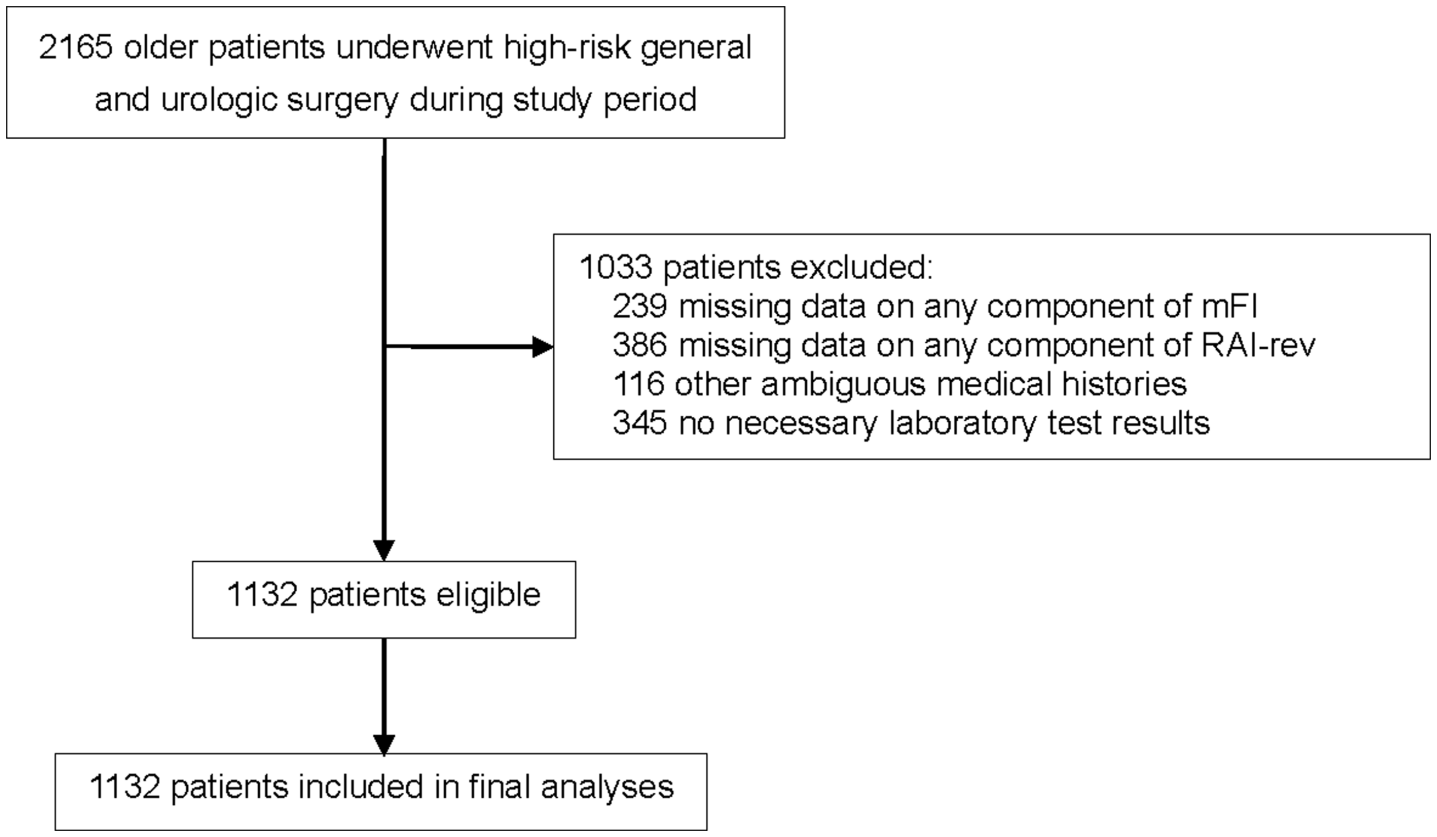
Flowchart of the study. MFI, modified frailty index; RAI-rev, revised- Risk Analysis Index.

From January 2018 to December 2020, 2,165 older patients (≥65 years of age) who experienced elective high-risk general and urologic surgical procedures were screened. Of these, 1,033 patients were excluded because they met the exclusion criteria (missing or ambiguous data on the components of mFI or RAI-rev, other ambiguous medical histories, or absence of necessary laboratory test results), leaving 1,132 patients in our analysis cohort (Figure 1).

**Figure 2:**
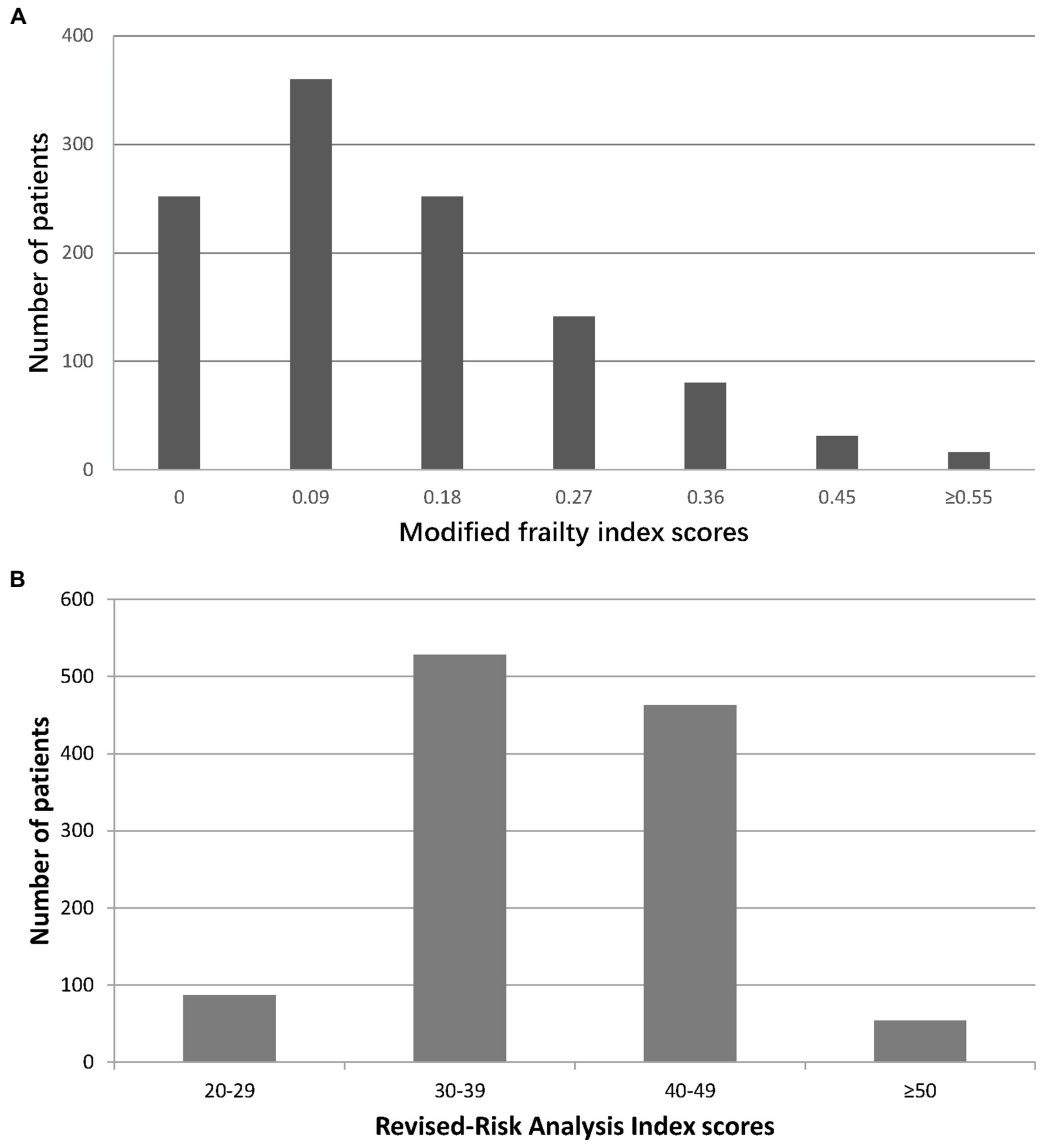
Distribution of frailty scores in the study cohort. **(A)** Modified frailty index; **(B)** Revised-Risk Analysis Index.

The study population had a mean age of 73.4 ± 6.2 years; 63.9% (723/1132) were male. The median mFI and RAI-rev values of our cohort were 0.09 [IQR: 0.09–0.18] and 38 [IQR: 37–43], respectively. The distribution of the mFI and RAI-rev scores across the cohort is displayed in Figure 2. According to the mFI score cutoff of 0.27 or higher, 268 (23.7%) patients were classified as frail. Based on the RAI-rev value cutoff of 45 or greater, 251 (22.2%) patients were identified as frail. During surgery, 1,040 (91.9%) patients underwent high-stress procedures, and 92 (8.1%) experienced very high-stress procedures (Table 1; Supplementary Table S1). After surgery, 107 patients (9.5%) developed postoperative life-threatening morbidity and death during hospitalization, of whom 94 (8.3%) and 13 (1.1%) developed CD IV complications and death, respectively (Table 1; Supplementary Table S4). Other baseline and perioperative characteristics are reported in Table 1.

**Table 1:** Baseline and perioperative characteristics.

	All patients (*n* = 1,132)	Without life-threatening morbidity and mortality (*n* = 1,025)	With life-threatening morbidity and mortality (*n* = 107)	*p* value
**Baseline characteristics**				
Age (years)	73.4 ± 6.2	73.2 ± 6.1	75.0 ± 6.1	**0.005**
Body mass index				**0.003**
<18.5 kg m^−2^	68 (6.0%)	54 (5.3%)	14 (13.1%)	
18.5–23.9 kg m^−2^	566 (50.0%)	522 (50.9%)	44 (41.1%)	
≥24 kg m^−2^	498 (44.0%)	449 (43.8%)	49 (45.8%)	
Modified frailty index scores	0.09 [0.09–0.18]	0.09 [0.09–0.18]	0.18 [0.09–0.36]	**0.001**
Frailty identified by mFI of ≥0.27	268 (23.7%)	227 (22.1%)	41 (38.3%)	**<0.001**
Hypertension	558 (49.3%)	497 (48.5%)	61 (57.0%)	0.093
Coronary heart disease	206 (18.2%)	174 (17.0%)	32 (29.9%)	**0.001**
Peripheral vascular disease	142 (12.5%)	126 (12.3%)	16 (15.0%)	0.429
Diabetes mellitus	316 (27.9%)	275 (26.8%)	41 (38.3%)	**0.012**
COPD or current pneumonia	94 (8.3%)	81 (7.9%)	13 (12.1%)	0.130
CHF exacerbation within 30d	11 (1.0%)	6 (0.6%)	5 (4.7%)	**<0.001**
Myocardial infarction within 6 months	5 (0.4%)	3 (0.3%)	2 (1.9%)	0.073
Previous stroke	193 (17.0%)	165 (16.1%)	28 (26.2%)	**0.008**
Stroke with deficits	57 (5.0%)	53 (5.2%)	4 (3.7%)	0.519
Functional dependence	275 (24.3%)	238 (23.2%)	37 (34.6%)	**0.009**
Acutely impaired sensorium^a^	3 (0.3%)	2 (0.2%)	1 (0.9%)	0.258
Revised-Risk Analysis Index scores	38 [37–43]	38 [37–43]	41 [37–45]	**<0.001**
Frailty identified by RAI-rev of ≥45	251 (22.2%)	212 (20.7%)	39 (36.4%)	**<0.001**
Male sex	723 (63.9%)	648 (63.2%)	75 (70.1%)	0.159
Age				**0.037**
65–69	366 (32.3%)	343 (33.5%)	23 (21.5%)	
70–74	304 (26.9%)	278 (27.1%)	26 (24.3%)	
75–79	260 (23.0%)	229 (22.3%)	31 (29.0%)	
80–84	144 (12.7%)	124 (12.1%)	20 (18.7%)	
≥85	58 (5.1%)	51 (5.0%)	7 (6.5%)	
Cancer	1,006 (88.9%)	915 (89.3%)	91 (85.0%)	0.186
Weight loss^b^	260 (23.0%)	231 (22.5%)	29 (27.1%)	0.285
Poor appetite	358 (31.6%)	305 (29.8%)	53 (49.5%)	**<0.001**
Renal failure	11 (1.0%)	8 (0.8%)	3 (2.8%)	**0.130**
Congestive heart failure	11 (1.0%)	6 (0.6%)	5 (4.7%)	**<0.001**
Shortness of breath	6 (0.5%)	3 (0.3%)	3 (2.8%)	**0.013**
Residence other than independent living	7 (0.6%)	3 (0.3%)	4 (3.7%)	**0.002**
Cognitive decline	20 (1.8%)	17 (1.7%)	3 (2.8%)	0.638
Alzheimer’s disease	5 (0.4%)	4 (0.4%)	1 (0.9%)	0.392
Vascular dementia	11 (1.0%)	10 (1.0%)	1 (0.9%)	>0.999
Parkinson’s disease	8 (0.7%)	6 (0.6%)	2 (1.9%)	0.170
Functional status				**0.002**
Independent	857 (75.7%)	787 (76.8%)	70 (65.4%)	
Partially dependent	263 (23.2%)	230 (22.4%)	33 (30.8%)	
Totally dependent	12 (1.1%)	8 (0.8%)	4 (3.7%)	
ASA classification				**<0.001**
I/II	629 (55.6%)	592 (57.8%)	37 (34.6%)	
III	458 (40.5%)	405 (39.5%)	53 (49.5%)	
IV	45 (4.0%)	28 (2.7%)	17 (15.9%)	
Current smoking^c^/quit ≤7 days	137 (12.1%)	124 (12.1%)	13 (12.1%)	0.987
Current alcoholism^d^	64 (5.7%)	56 (5.5%)	8 (7.5%)	0.391
Severe arrhythmia^e^	92 (8.1%)	80 (7.8%)	12 (11.2%)	0.219
Asthma	22 (1.9%)	20 (2.0%)	2 (1.9%)	>0.999
Mental disorders^f^	29 (2.6%)	27 (2.6%)	2 (1.9%)	0.877
Visual/hearing impairment	47 (4.2%)	43 (4.2%)	4 (3.7%)	>0.999
Chronic hepatic dysfunction^g^	60 (5.3%)	51 (5.0%)	9 (8.4%)	0.131
Chronic corticosteroid therapy^h^	41 (3.6%)	38 (3.7%)	3 (2.8%)	0.838
Hyper−/hypothyroidism	29 (2.6%)	27 (2.6%)	2 (1.9%)	0.877
Anemia^i^	376 (33.2%)	334 (32.6%)	42 (39.3%)	0.164
Blood coagulation disorder	15 (1.3%)	12 (1.2%)	3 (2.8%)	0.336
Dyslipidemia	614 (54.2%)	554 (54.0%)	60 (56.1%)	0.689
Hypoalbuminemia				**<0.001**
None	619 (54.7%)	576 (56.2%)	43 (40.2%)	
30.0–39.9 g l^−1^	460 (40.6%)	408 (39.8%)	52 (48.6%)	
<30.0 g l^−1^	53 (4.7%)	41 (4.0%)	12 (11.2%)	
Na^+^ < 135.0 mmol l^−1^	91 (8.0%)	74 (7.2%)	17 (15.9%)	**0.002**
**Intraoperative data**				
Risk stratification of surgery by OSS^j^				**0.002**
High stress	1,040 (91.9%)	950 (92.7%)	90 (84.1%)	
Very high stress	92 (8.1%)	75 (7.3%)	17 (15.9%)	
Duration of surgery (min)	237 [190–297]	231 [188–292]	256 [195–318]	**0.006**
Type of anesthesia				0.701
General	488 (43.1%)	440 (42.9%)	48 (44.9%)	
Combined regional-general	644 (56.9%)	585 (57.1%)	59 (55.1%)	
Estimated blood loss (ml)	100 [50–200]	100 [50–200]	150 [100–300]	**0.008**
Blood transfusion	130 (11.5%)	114 (11.1%)	16 (15.0%)	0.237
**Postoperative outcomes**				
CD grade IV	94 (8.3%)	**−**	94 (87.9%)	**−**
Death	13 (1.1%)	**−**	13 (12.1%)	**−**
ICU admission	344 (30.4%)	258 (25.2%)	86 (80.4%)	**<0.001**
Prolonged hospital stay^k^	378 (33.4%)	294 (28.7%)	84 (78.5%)	**<0.001**
Adverse discharge destination^l^	33 (2.9%)	9 (0.9%)	24 (22.4%)	**<0.001**

Data are *n* (%), mean ± SD, or median [IQR]. *p*-values were derived from comparing the patients with life-threatening morbidity and mortality and those without. *p* values in bold indicate <0.05. COPD, chronic obstructive pulmonary disease; CHF, congestive heart failure; ASA, American Society of Anesthesiologists; Na^+^, serum natremia concentration; OSS, operative stress score; CD, Clavien**-**Dindo classification of complication; ICU, intensive care unit.

a Refers to acute mental status changes and/or delirium in the context of the current illness. Patients with chronic or long-standing mental status changes secondary to chronic mental illness or chronic dementing illnesses are not included.

b Unintentional weight loss ≥10% from baseline within 6 months, or ≥5% within 3 months, or ≥2% within 1 month.

c Smoking refers to daily smoking of cigarettes up to half a pack for at least 2 years.

d Alcoholism refers to ethanol consumption ≥40 g/d for men and ≥20 g/d for women, lasting for more than 5 years. Ethanol (g) = alcohol consumption (ml) × ethanol content (%) × 0.8.

e Includes atrial fibrillation, frequent (>6 beats/min) or multifocal ventricular premature beat, paroxysmal supraventricular tachycardia, second/third-degree atrioventricular block, and sick sinus syndrome.

f Include diagnosed depression, anxiety, schizophrenia, phobia, and hallucination.

g Defined as Child-Pugh class B and C.

h With a duration of >1 month.

i Diagnosed according to the hemoglobin values from the last laboratory test before surgery, male: <120 g l^−1^, female: <110 g l^−1^_._

j Identified the risk stratification of surgery by physiologic stress, i.e., operative stress score (OSS). The surgical procedures in the study were those with OSS level 4 (i.e., high stress) and OSS level 5 (i.e., very high stress) (23). Detailed data on surgery procedures is provided in Supplemental Table S1.

k Defined as greater than the 75th percentile of the length of hospital for each type of surgery.

l Defined as discharge to destinations other than home (e.g., skilled care facility or other hospitals).

### Postoperative outcomes according to frailty

Compared with patients with an mFI of <0.27, those with an mFI of ≥0.27 had a higher rate of the composite primary outcome or life-threatening morbidity, had a shorter time to develop the life-threatening morbidity and mortality, had more postoperative ICU admissions, stayed longer in hospital, and experienced more adverse discharge destinations (All *p* < 0.05). Similarly, there were significant differences in the above outcomes between the patients with an RAI-rev score of <45 and those with a score of ≥45 (all *p* < 0.05; Table 2). In addition, we observed that the patients with an RAI-rev score of ≥45 developed more in-hospital death than those with a score of <45 (3.6% vs. 0.5%, *p* < 0.001); whereas we found no significant difference in mortality between the two mFI subgroups (1.9% vs. 0.9%, *p* = 0.351; Table 2).

**Table 2:** Postoperative outcomes according to frailty.

	Modified frailty index			Revised-Risk Analysis Index		
	<0.27 (*n* = 864)	≥0.27 (*n* = 268)	*P* value	<45 (*n* = 881)	≥45 (*n* = 251)	*p* value
The primary outcome	66 (7.6%)	41 (15.3%)	**<0.001**	68 (7.7%)	39 (15.5%)	**<0.001**
Life-threatening morbidity	58 (6.7%)	36 (13.4%)	**<0.001**	64 (7.3%)	30 (12.0%)	**0.018**
Mortality	8 (0.9%)	5 (1.9%)	0.351	4 (0.5%)	9 (3.6%)	**<0.001**
Time to life-threatening morbidity and mortality (day)^a^	27.826 (27.289–28.363)	25.567 (24.301–26.833)	**<0.001**	27.780 (27.230–28.330)	25.506 (24.209–26.802)	**<0.001**
Postoperative ICU admission	215 (24.9%)	129 (48.1%)	**<0.001**	231 (26.2%)	113 (45.0%)	**<0.001**
Prolonged hospital stay	249 (28.8%)	129 (48.1%)	**<0.001**	266 (30.2%)	112 (44.6%)	**<0.001**
Adverse discharge destination	20 (2.3%)	13 (4.9%)	**0.031**	17 (1.9%)	16 (6.4%)	**<0.001**

Data are *n* (%) or mean [95% CI]. *p* values in bold indicate <0.05. ICU, intensive care unit.

a Analyzed with Kaplan–Meier survival analysis (log-rank test).

### Correlation and agreement between the two frailty tools

Overall the Spearman’s correlation coefficient for the continuous scores was 0.243 (*p* < 0.001), indicating a low correlation between the two frailty indices. The overall percentage of agreement between the two dichotomized measures was 72.2% (817/1132). Specifically, 102 (9.0%) and 715 (63.2%) patients were identified as frail and non-frail by both measures, respectively. Cohen’s Kappa test showed slight agreement between the two dichotomized measures (Cohen’s Kappa coefficient: 0.213, *p* < 0.001; Table 3).

**Table 3:** Two way cross-tabulation of dichotomized mFI and RAI-rev measures.

	Modified frailty index		
	Non-frail	Frail	Total
Revised-Risk Analysis Index			
Non-frail	715 (63.2%)	166 (14.7%)	881 (77.8%)
Frail	149 (13.2%)	102 (9.0%)	251 (22.2%)
Total	864 (76.3%)	268 (23.7%)	1,132 (100%)

Data are *n* (%). The overall percentage of agreement between the two dichotomized frailty measures was 72.2%. Cohen’s Kappa coefficient was 0.213 (*P* < 0.001).

### Association between mFI and life-threatening morbidity and mortality

The univariate logistic regression analysis showed that the odds of life-threatening morbidity and mortality increased by 34.5% with every unit (i.e., 0.09 points) increase in the mFI score (unadjusted OR 1.345 per 0.09-point increase in score, 95% CI 1.183–1.528, *p* < 0.001; Supplementary Table S5). After adjusting for confounding factors (i.e., age, body mass index, renal failure, hypoalbuminemia, hyponatremia, and risk stratification and duration of surgery), the rising mFI score remained to be significantly associated with an increased risk of life-threatening morbidity and mortality (adjusted OR 1.319 per 0.09-point increase in score, 95% CI 1.151–1.511, *p* < 0.001; Table 4; Supplementary Table S6).

**Table 4:** Association of frailty with life-threatening morbidity and mortality (logistic regression analyses).

	Univariate analyses		Multivariable analyses	
	Unadjusted OR (95% CI)	*p* value	Adjusted OR (95% CI)	*p* value
Modified frailty index scores^a^	1.345 (1.183–1.528)	<0.001	1.319 (1.151–1.511)	<0.001
Frailty based on mFI of ≥0.27^a^	2.184 (1.440–3.313)	<0.001	2.059 (1.328–3.193)	0.001
Revised-Risk Analysis Index scores^b^	1.066 (1.032–1.100)	<0.001	1.052 (1.018–1.087)	0.002
Frailty based on RAI-rev of ≥45^b^	2.199 (1.443–3.353)	<0.001	1.862 (1.188–2.919)	0.007

OR, odds ratio; CI, confidence interval; mFI, modified frailty index; RAI-rev, revised-Risk Analysis Index.

a After testing for multicollinearity, mFI (as continuous or dichotomous variable) and other factors with *p* values <0.10 in univariate logistic regression analyses (including age, body mass index, renal failure, hypoalbuminemia, hyponatremia, and risk stratification and duration of surgery) were included in the multivariable logistic regression model to identify the adjusted association between high mFI (per 0.09-point increase in mFI score or mFI of ≥0.27) and the primary outcome. The 11 variables covered by mFI were not separately enrolled in multivariable analyses. See Supplementary Table S6 for details.

b After testing for multicollinearity, RAI-rev (as continuous or dichotomous variable) and other factors with *p* values <0.10 in univariate logistic regression analyses (including body mass index, hypertension, coronary heart disease, previous stroke, diabetes mellitus, hypoalbuminemia, hyponatremia, and risk stratification and duration of surgery) were included in the multivariable logistic regression model to identify the adjusted association between high RAI-rev (per 1-point increase in RAI-rev score or RAI-rev of ≥45) and the primary outcome. The 11 variables covered by the RAI-rev were not separately enrolled in multivariable analyses. See Supplementary Table S7 for details.

Frailty, identified by mFI scores of ≥0.27, was associated with an increased risk of the primary outcome in both univariate analysis (unadjusted OR 2.184, 95% CI 1.440–3.313, *p* < 0.001; Supplementary Table S5) and multivariable analysis (adjusted OR 2.059, 95% CI 1.328–3.193, *p* = 0.001) after correcting for the above confounding factors (Table 4; Supplementary Table S6).

### Association between RAI-rev and life-threatening morbidity and mortality

The univariate logistic regression analysis demonstrated that rising RAI-rev score was related to increased odds of life-threatening morbidity and mortality (unadjusted OR 1.066 per 1-point increase in score, 95% CI 1.032–1.100, *p* < 0.001; Supplementary Table S5). After correcting for confounding factors (i.e., body mass index, hypertension, coronary heart disease, previous stroke, diabetes mellitus, hypoalbuminemia, hyponatremia, and risk stratification and duration of surgery), the rising RAI-rev score remained to be an independent predictor of life-threatening morbidity and mortality (adjusted OR 1.052 per 1-point increase in score, 95% CI 1.018–1.087, *p* = 0.002; Table 4; Supplementary Table S7).

Frailty, based on RAI-rev scores of ≥45, predicted the primary outcome in both univariate analysis (unadjusted OR 2.199, 95% CI 1.443–3.353, *p* < 0.001; Supplementary Table S5) and multivariable analysis (adjusted OR 1.862, 95% CI 1.188–2.919, *p* = 0.007) after adjustment for the above confounding factors (Table 4; Supplementary Table S7).

### Time effect of frailty on 30-day life-threatening morbidity and mortality

In the Kaplan–Meier analysis, when compared with non-frail (identified by mFI scores of <0.27) patients, the frail patients (determined by mFI scores of ≥0.27) had a shortened time to develop 30-day life-threatening morbidity and mortality (log-rank test: *p* < 0.001; Table 2; Supplementary Figure S1). Similar results were observed when frailty was diagnosed by RAI-rev scores of ≥45 (log-rank test: *p* < 0.001; Table 2; Supplementary Figure S2).

Multivariable Cox proportional hazards regression analyses demonstrated that frailty identified by mFI scores of ≥0.27 was associated with a 2-fold increased hazard of developing 30-day life-threatening morbidity and mortality (adjusted HR 2.042, 95% CI 1.353–3.083, *p* = 0.001; Supplementary Table S8). Similarly, frailty diagnosed by RAI-rev scores of ≥45 was linked with a 1.8-fold higher hazard of 30-day life-threatening morbidity and mortality (adjusted HR 1.822, 95% CI 1.198–2.770, *p* = 0.005; Supplementary Table S9).

### Predictive performances of mFI and RAI-rev in predicting life-threatening morbidity and mortality

**Figure 3:**
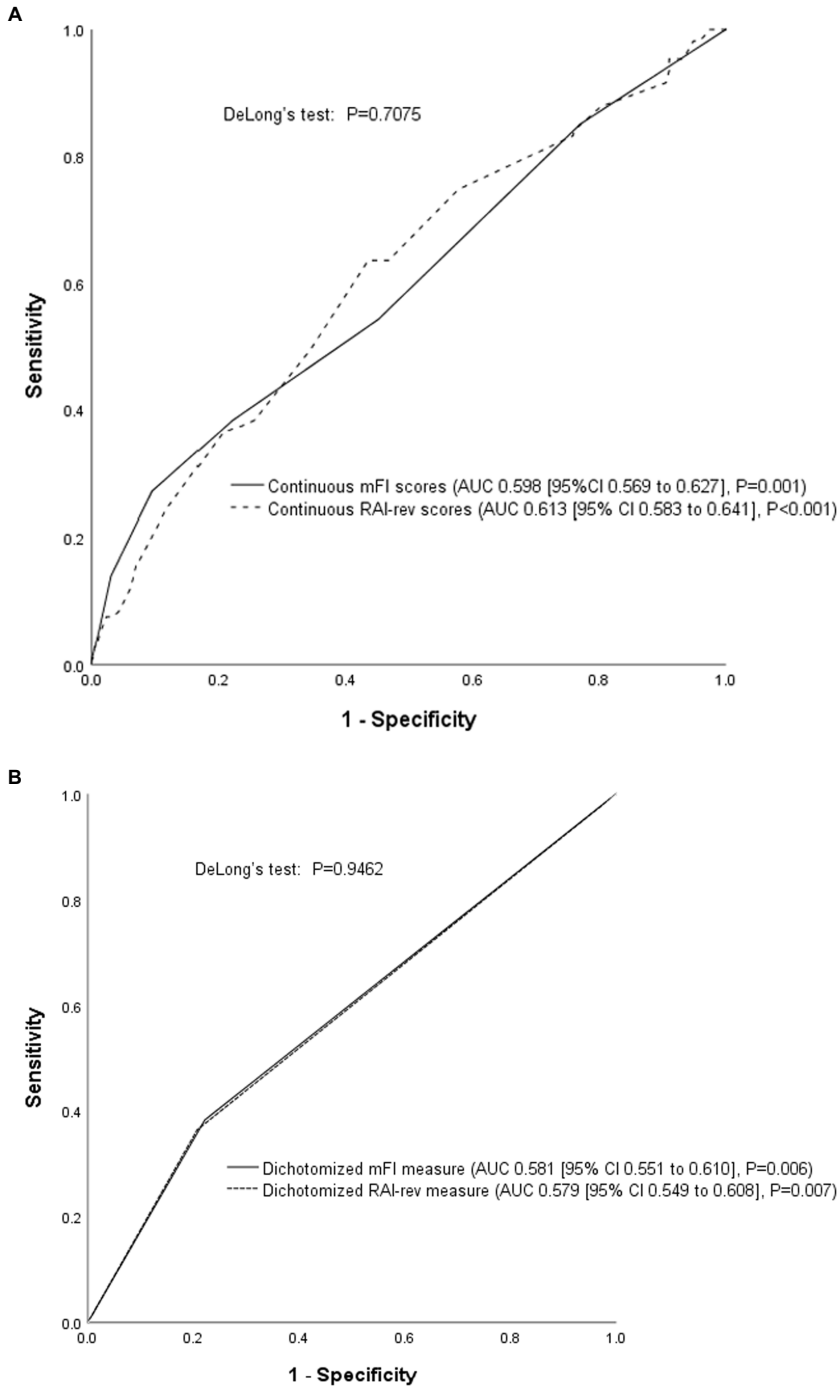
Receiver operating characteristic (ROC) curve analysis. **(A)** Comparison of the continuous mFI and RAI-rev scores; **(B)** Comparison of dichotomized mFI and RAI-rev measures. ROC curve analyses showed that the performances of mFI and RAI-rev were poor and comparable in predicting life-threatening morbidity and mortality. MFI, modified frailty index; RAI-rev, revised-Risk Analysis Index.

The AUCs of continuous mFI and RAI-rev scores in predicting life-threatening morbidity and mortality were 0.598 (95% CI 0.569–0.627) and 0.613 (95% CI 0.583–0.641), respectively. Although the AUC of RAI-rev was slightly higher than that of mFI, no statistical difference between them was detected (DeLong’s test: Z = 0.375, *p* = 0.7075; Figure 3).

As seen in Figure 3, the performances of dichotomized mFI (0.581 [95% CI 0.551–0.610]) and RAI-rev (0.579 [95% CI 0.549–0.608]) measures were also comparable in predicting the primary outcome (DeLong’s test: Z = 0.0675, *p* = 0.9462).

## Discussion

This retrospective cohort study determined that rising mFI and RAI-rev scores were associated with a higher risk of life-threatening morbidity and mortality in older patients after elective high-risk abdominal surgery. However, both the two frailty indices performed poor discriminative abilities for the occurrence of life-threatening morbidity and mortality.

In the present study, we found a low correlation and slight agreement between the two frailty indices. As shown in Table 3, among the 268 patients identified as frail by mFI, only 38% (102/268) were also diagnosed with frailty by RAI-rev; meanwhile, only 41% (102/251) of the RAI-rev frail patients were classified as frail by mFI. This finding was unsurprising since the two tools shared limited overlap between frailty spectrums and assigned different weights to components. The selection of cutoff values also affected the agreement between the two frailty indices. The slight agreement between them indicates the potential for combining the two measures to capture more useful patient-level information, which provides clues for further exploration.

The effect of frailty on major postoperative morbidity and mortality has been extensively studied (5–11, 14, 17, 23, 24, 27). In a retrospective cohort study of 9,986 adult patients receiving pancreaticoduodenectomy, Mogal et al. (10) determined that increased mFI scores (≥0.27) predicted a 1.54-fold elevated risk of major complications or 30-day mortality. In the current study, we identified a stronger association between high mFI (≥0.27) and serious morbidity and mortality (adjusted OR: 2.06), which could be mainly attributed to the fact that our patients were older and performed worse baseline status (e.g., higher prevalence of functional dependence) than those in the above study. In another observational study of ambulatory patients undergoing minor surgery, Shah et al. (7) examined the relationship of RAI-sev with 1-year mortality and found that frailty (RAI-rev score: 45–52) and severe frailty (RAI-rev score: ≥53) were associated with hazard ratios of 2.76 and 4.83 for mortality (compared with normal status, i.e., RAI-rev score: 30–36), respectively. However, the above results were not corrected for any confounding factor. As far as we know, no previous studies have estimated the adjusted effects of rising RAI-sev score on the occurrence of serious morbidity and mortality after elective high-risk surgery. Our study filled this knowledge gap and expanded the existing evidence. Our findings highlighted the importance and urgent need to augment the application of routine frailty screening before surgery in older populations.

In the present study, ROC analysis results demonstrated that neither the mFI nor the RAI-rev was equipped with good discrimination for serious morbidity and mortality in older patients undergoing elective high-risk abdominal surgery. Our findings are congruent with previous studies that applicated frailty indices to predict postoperative morbidity (8, 9, 27). The poor discriminative abilities of the two frailty measures for postoperative morbidity might be explained by the following three reasons. First, the mFI fails to cover multiple frailty spectrums because it evaluates only two domains (comorbidity burden and functional impairment). Although the RAI-rev captures multiple frailty features, some of its elements are typically representative of acute disease processes, which are infrequent among elective high-risk surgery candidates. For example, the prevalence of congestive heart failure or shortness of breath was quite low in our cohort. Second, surgeons are always more cautious to determine the surgical candidacy of a patient when considering the upcoming procedure as high risk. In most cases, the patient assessed as too frail may turn to conservative treatment instead of receiving aggressive high-risk surgery. Thus, the exclusion of severely frail patients might limit the predictive performance of frailty in this study cohort. Indeed, existing data suggest that the association between frailty and adverse outcomes is stronger in low-risk surgery than in high-risk surgery (23, 28, 29). Third, the etiology of postoperative morbidity is multifactorial, and the patient-level risk factors alone cannot adequately account for the variation in complication risk. Further studies should consider the combination of frailty with additional risk variables, such as other baseline characteristics and surgical-related risk factors, to predict the risk of postoperative morbidity.

Our findings demonstrated clinical significance and might play an important role in perioperative settings. Based on the multivariable logistic and Cox regression analysis results, frailty was significantly associated with a higher risk of life-threatening morbidity and mortality in older patients after elective high-risk abdominal surgery. This finding can help clinicians forecast the elevated risk of serious postoperative outcomes in frail older patients and improve the shared decision-making process. Once a patient is screened as frail, determining the goals of care and selecting the optimal approach to achieve the goals of care constitute crucial components of shared decision-making. It should be carefully considered whether aggressive surgical intervention or palliative care can get frail patients to their goals of care. Realistic expectations and appropriate decision-making may, in turn, decrease perioperative mortality (30). Furthermore, this finding can help guide the more efficient allocation of scarce perioperative care resources to high-risk patients, such as necessary postoperative ICU admission and active application of advanced invasive or non-invasive monitoring skills, thereby enhancing the safety and quality of high-risk surgery in older patients. Based on the ROC analysis results, the two frailty indices presented poor discriminative power in predicting the primary outcome. Despite this, the above finding generates clues for further research. It is anticipated that the combination of frailty with other baseline and perioperative risk factors may emerge as a potentially useful model to predict serious morbidity. Additionally, larger studies with the recruitment of more patients with severe frailty are needed to draw more reliable conclusions.

Besides the retrospective nature, our study had several notable limitations. First, as mentioned above, the patients with severe frailty were inevitably excluded from the study cohort due to the high-risk nature of the surgery, which might lead to selection bias. Second, the primary outcome was limited to in-hospital serious morbidity and mortality. The complications below CD grade IV were not included in our analysis and the post-discharge outcomes were not gathered, which might underestimate the rate of adverse outcomes. Finally, in our study, the composite endpoint rate was fairly low, especially mortality. Our relatively limited sample size could not enable us to fully elucidate the relationship between frailty and mortality. Despite these, our results demonstrate clinical significance and generate clues for further investigation.

## Conclusion

In conclusion, this study determined that high mFI and RAI-rev scores were associated with an increased risk of life-threatening morbidity and mortality in older patients following elective high-risk abdominal surgery. However, both frailty indices displayed poor discrimination for the composite outcome of life-threatening morbidity and mortality.

## Data availability statement

The raw data supporting the conclusions of this article will be made available by the authors, without undue reservation.

## Ethics statement

The studies involving human participants were reviewed and approved by The Biomedical Research Ethics Committee of Peking University First Hospital. The ethics committee waived the requirement of written informed consent for participation.

## Author contributions

C-QL contributed to the conception, study design, and supervision. C-QL, HK, and Z-ZX collected data. C-QL, J-HM, and X-YL analyzed and interpreted the data. C-QL drafted the manuscript. All authors critically revised the manuscript, agreed to submit it to the current journal, and gave final approval of the version to be published.

## Conflict of interest

The authors declare that the research was conducted in the absence of any commercial or financial relationships that could be construed as a potential conflict of interest.

## Publisher’s note

All claims expressed in this article are solely those of the authors and do not necessarily represent those of their affiliated organizations, or those of the publisher, the editors and the reviewers. Any product that may be evaluated in this article, or claim that may be made by its manufacturer, is not guaranteed or endorsed by the publisher.
