# Supplementary material for: Comparison of two frailty indices in predicting life-threatening morbidity and mortality among older patients undergoing elective high-risk abdominal surgery
Source: Front Public Health. 2023 Apr 5;11:1055001. doi: 10.3389/fpubh.2023.1055001 (PMC10113537; doi:10.3389/fpubh.2023.1055001)
Supplement: Supplementary file 1 [file Data_Sheet_1.docx]

Supplementary Material

**Supplementary Table 1** Surgical procedures stratified according to Operative Stress Score [23]

| Category, Procedure Type | All patients (*n*=1132) |
| --- | --- |
| **Operative Stress Score 4, High Stress** |  |
| All | 1040 (91.9%) |
| Colectomy, open | 115 (10.2%) |
| Gastrectomy | 196 (17.3%) |
| Hepatectomy, resection of liver; partial lobectomy | 73 (6.4%) |
| Proctectomy, laparoscopic or open | 304 (26.9%) |
| Pancreatectomy, distal subtotal, with or without splenectomy; without pancreatojejunostomy | 28 (2.5%) |
| Excision of huge primary retroperitoneal tumor | 22 (1.9%) |
| Nephrectomy, partial | 38 (3.4%) |
| Nephrectomy, radical, with vena caval thrombectomy | 8 (0.7%) |
| Pheochromocytoma | 19 (1.7%) |
| Complete cystectomy, with continent diversion, including using a  segment of ileum to construct neobladder, or cutaneous ureterostomy | 217 (19.2%) |
| Prostatectomy, retropubic radical, with bilateral pelvic lymphadenectomy | 20 (1.8%) |
| **Operative Stress Score 5, Very High Stress** |  |
| All | 92 (8.1%) |
| Pancreaticoduodenectomy (Whipple-type procedure) | 92 (8.1%) |

Data are *n* (%).

**Supplementary Table 2** Modified Frailty Index [5]

| Hypertension requiring medication |
| --- |
| Diabetes mellitus treated with or without insulin therapy |
| Chronic obstructive pulmonary disease or current pneumonia |
| History of congestive heart failure exacerbation within 30 days |
| History of myocardial infarction within 6 months |
| History of angina within 1 month before surgery or any percutaneous coronary intervention or coronary artery bypass grafting |
| Transient ischemic attack or cerebrovascular accident without deficits |
| Cerebrovascular accident with deficits |
| Peripheral vascular disease, rest pain, or gangrene |
| Acutely impaired sensorium ^a^ |
| Partially or totally dependent functional status before surgery |

Total score (range): 0 to 1

^a^ Refers to acute mental status changes and/or delirium in the context of the current illness. Patients with chronic or long-standing mental status changes secondary to chronic mental illness or chronic dementing illnesses are not included.

**Supplementary Table 3** Revised-Risk Analysis Index Scoring system [6]

| Variable | RAI-rev | |
| --- | --- | --- |
| Male sex | 3 | |
| Weight loss | 4 | |
| Poor appetite | 4 | |
| Renal failure | 8 | |
| Congestive heart failure | 5 | |
| Shortness of breath at rest | 3 | |
| Residence other than independent living | 1 | |
| Age* cancer | Without cancer | With cancer |
| Age |  |  |
| 65–69 | 20 | 34 |
| 70–74 | 22 | 34 |
| 75–79 | 24 | 35 |
| 80–84 | 26 | 35 |
| 85–89 | 28 | 36 |
| 90–94 | 30 | 36 |
| 95–99 | 32 | 37 |
| ADL* cognitive decline | Without cognitive decline | With cognitive decline |
| Totally dependent | 14 | 16 |
| Partially dependent | 7 | 11 |
| Independent | 0 | 5 |

Total score (range): 0 to 81

ADL, activities of daily living.

**Supplementary Table 4** Individual life-threatening morbidity

| Specific morbidity | All patients (*n*=1132) |
| --- | --- |
| Cardiovascular morbidity, *n* (%) | 67 (6.0%) |
| Acute coronary syndrome ^a^ | 24 (2.1%) |
| New-onset severe arrhythmia ^b^ | 12 (1.1%) |
| Circulatory insufficiency ^c^ | 34 (3.0%) |
| Acute heart failure or exacerbation of congestive heart failure ^d^ | 18 (1.6%) |
| Cardiac arrest ^e^ | 4 (0.4%) |
| Respiratory morbidity, *n* (%) | 47 (4.2%) |
| Respiratory failure ^f^ | 44 (3.9%) |
| Bronchospasm ^g^/asthma attack ^h^ | 3 (0.3%) |
| Neurological morbidity, *n* (%) | 11 (1.0%) |
| Stroke ^i^ | 11 (1.0%) |
| Renal morbidity, *n* (%) | 18 (1.6%) |
| Acute renal failure ^j^ | 18 (1.6%) |
| Thrombotic morbidity, *n* (%) | 13 (1.1%) |
| Pulmonary embolism ^k^ | 4 (0.4%) |
| Disseminated intravascular coagulation ^l^ | 9 (0.8%) |
| Surgery-related morbidity, *n* (%) | 75 (6.6%) |
| Intra-abdominal/pelvic abscess ^m^ | 32 (2.8%) |
| Anastomotic leak ^n^ | 24 (2.1%) |
| Ileus ^o^ | 14 (1.2%) |
| Surgical hemorrhage ^p^ | 12 (1.1%) |
| Infectious morbidity, *n* (%) | 66 (5.8%) |
| Sepsis ^q^ | 66 (5.8%) |

Data are *n* (%).

^a^ Includes acute myocardial infarction and unstable angina, which were confirmed by clinical symptoms, electrocardiographic changes, imaging evidence, serum cardiac troponin I concentration, and requirement of IC/ICU management.

^b^ Confirmed by 12-lead electrocardiogram and necessitated IC/ICU level management.

^c^ Requiring inotropics or vasopressors for more than 24 hours after surgery and IC/ICU management.

^d^ Diagnosed by the presence of new-onset orthopnea with evidence of fluid retention (i.e., elevated jugular venous pressure, evidence of pulmonary edema, and/or peripheral edema), an elevated plasma brain natriuretic peptide of above 400 pg ml^-1^, and necessitated IC/ICU management.

^e^ The absence of large artery pulsation and heart sound, subsequent loss of consciousness, respiratory arrest, dilated pupils and even death, caused by the sudden stop of heart beating.

^f^ *P*aO_2_ <60 mmHg on room air, a ratio of *P*aO_2_ to inspired oxygen fraction <300, or arterial oxyhemoglobin saturation measured with pulse oximetry <90% and requiring IC/ICU management, or intubation and mechanical ventilation.

^g^ Confirmed by new-onset expiratory wheezing, necessitated treatment with intubation and mechanical ventilation or IC/ICU management.

^h^ Diagnosed according to clinical signs, necessitated intubation and mechanical ventilation, or IC/ICU management.

^i^ Persisted new focal neurologic deficit confirmed by neurologic imaging, requiring IC/ICU level care.

^j^ New-onset renal failure that required renal replacement therapy.

^k^ Hypotension or shock suspected of pulmonary embolism requiring IC/ICU level management and meeting one of the following: filling defect in any branch of the pulmonary artery in computed tomographic pulmonary angiogram or right ventricular overload in the echocardiogram.

^l^ Diagnosed by abnormal bleeding symptoms and more than three anomalies in the following items: platelet <100×10^9^ l^-1^ or progressive decline; fibrinogen <1.5 g l^-1^ or progressive decline or >4 g l^-1^; plasma fibrin degradation product (FDP) >20 mg l^-1^ or D-dimer level increased or positive, or 3P test (plasma protamine paracoagulation test) positive; prothrombin time (PT) shorter or longer than 3 s or activated partial thromboplastin time (APTT) shorter or longer than 10 s.

^m^ Intra-abdominal/pelvic abscess (excluding those caused by anastomotic leak) that caused sepsis or at least one organ failure.

^n^ Anastomotic leak that caused sepsis or at least one organ failure.

^o^ Extensive intestinal necrosis or ileus that caused sepsis or at least one organ failure.

^p^ Postoperative surgical bleeding that caused severe hemodynamic instability and required IC/ICU level management.

^q^ Two or more criteria of systemic inflammatory response syndrome, with known infection and new-onset dysfunction of at least one organ/system.

**Supplementary Table 5** Factors in association with postoperative life-threatening morbidity and mortality ( univariate logistic regression analyses)

| Variables | Number | OR (95% CI) | *P* value |
| --- | --- | --- | --- |
| Age | 1132 | 1.045 (1.013 to 1.077) | **0.006** |
| Female | 409 | 0.733 (0.476 to 1.131) | 1.160 |
| Body mass index |  |  |  |
| 18.5-23.9 kg m^-2^ | 566 | Reference |  |
| <18.5 kg m^-2^ | 68 | 3.076 (1.584 to 5.972) | **0.001** |
| ≥24 kg m^-2^ | 498 | 1.295 (0.845 to 1.983) | 0.235 |
| Modified Frailty Index scores | 1132 | 1.345 (1.183-1.528) | **<0.001** |
| Frailty based on mFI of ≥0.27 | 268 | 2.184 (1.440 to 3.313) | **<0.001** |
| Hypertension | 558 | 1.409 (0.943 to 2.106) | **0.095** |
| Coronary heart disease | 206 | 2.087 (1.337 to 3.256) | **0.001** |
| Peripheral vascular disease | 142 | 1.254 (0.714 to 2.203) | 0.430 |
| Diabetes mellitus | 316 | 1.694 (1.120 to 2.562) | **0.012** |
| COPD or current pneumonia | 94 | 1.612 (0.865 to 3.005) | 0.133 |
| Previous stroke | 193 | 1.847 (1.164 to 2.933) | **0.009** |
| Stroke with deficits | 57 | 0.712 (0.253 to 2.008) | 0.521 |
| Revised-Risk Analysis Index scores | 1132 | 1.063 (1.030 to 1.098) | **<0.001** |
| Frailty based on RAI-rev of ≥45 | 251 | 2.199 (1.443 to 3.353) | **<0.001** |
| Malignant tumor | 1006 | 0.684 (0.388 to 1.205) | 0.189 |
| Recent weight loss ^a^ | 260 | 1.278 (0.814 to 2.006) | 0.286 |
| Renal failure | 11 | 3.667 (0.958 to 14.035) | **0.058** |
| Cognitive decline | 20 | 1.710 (0.493 to 5.933) | 0.398 |
| Current smoking ^b^ /quit ≤7 days | 137 | 1.005 (0.546 to 1.849) | 0.987 |
| Current alcoholism ^c^ | 64 | 1.398 (0.648 to 3.017) | 0.393 |
| Severe arrhythmia ^d^ | 92 | 1.492 (0.785 to 2.836) | 0.222 |
| Asthma | 22 | 0.957 (0.221 to 4.152) | 0.953 |
| Mental disorders ^e^ | 29 | 0.704 (0.165 to 3.002) | 0.635 |
| Visual/hearing impairment | 47 | 0.887 (0.312 to 2.520) | 0.822 |
| Chronic hepatic dysfunction ^f^ | 60 | 1.754 (0.838 to 3.670) | 0.136 |
| Chronic corticosteroid therapy ^g^ | 41 | 0.749 (0.227 to 2.469) | 0.635 |
| Hyper-/hypothyroidism | 29 | 0.704 (0.165 to 3.002) | 0.635 |
| Anemia ^h^ | 376 | 1.337 (0.888 to 2.013) | 0.165 |
| Blood coagulation disorder | 15 | 2.435 (0.676 to 8.768) | 0.173 |
| Dyslipidemia | 614 | 1.085 (0.727 to 1.621) | 0.689 |
| Hypoalbuminemia |  |  |  |
| None | 619 | Reference |  |
| 30.0–39.9 g l^-1^ | 460 | 1.707 (1.118 to 2.607) | **0.013** |
| <30.0 g l^-1^ | 53 | 3.921 (1.920 to 8.007) | **<0.001** |
| Na^+^ <135.0 mmol l^-1^ | 91 | 2.427 (1.373 to 4.291) | **0.002** |
| Risk stratification of surgery by OSS ^i^ |  |  |  |
| High stress | 1040 | Reference |  |
| Very high stress | 92 | 2.393 (1.354 to 4.227) | **0.003** |
| Duration of surgery (hour) | 1132 | 1.181 (1.044 to 1.336) | **0.008** |
| Type of anesthesia |  |  |  |
| General | 488 | Reference |  |
| Regional/combined regional-general | 644 | 0.925 (0.619 to 1.380) | 0.701 |
| Estimated blood loss (100 ml) | 1132 | 1.025 (0.983 to 1.069) | 0.254 |
| Intra-operative blood transfusion | 130 | 1.405 (0.798 to 2.747) | 0.239 |

*P* values in bold indicate <0.10.

MFI, modified frailty index; RAI-rev, revised Risk Analysis Index; COPD, chronic obstructive pulmonary disease; Na^+^, serum natremia concentration; OSS, operative stress score.

^a^ Unintentional weight loss ≥10% from baseline within 6 months, or ≥5% within 3 months, or ≥2 % within 1 month.

^b^ Smoking refers to daily smoking of cigarettes up to half a pack for at least two years.

^c^ Alcoholism refers to ethanol consumption ≥40 g/d for men and ≥20 g/d for women, lasting for more than 5 years. Ethanol (g) = alcohol consumption (ml) × ethanol content (%) × 0.8.

^d^ Includes atrial fibrillation, frequent (>6 beats/min) or multifocal ventricular premature beat, paroxysmal supraventricular tachycardia, second/third-degree atrioventricular block, and sick sinus syndrome.

^e^ Include diagnosed depression, anxiety, schizophrenia, phobia, and hallucination.

^f^ Refers to hepatic impairment classified as Child-Pugh class B and C.

^g^ With a duration of >1 month.

^h^ Diagnosed according to the hemoglobin values from the last laboratory test before surgery, male: <120 g l^-1^, female: <110 g l^-1^.

^i^ Identified the risk stratification of surgery by physiologic stress, i.e., operative stress score (OSS). The surgical procedures in the study were those with OSS level 4 (i.e., high stress) and OSS level 5 (i.e., very high stress) [23]. Detailed data on surgery procedures are provided in Supplemental Table 1.

**Supplementary Table 6** Association of mFI with postoperative life-threatening morbidity and mortality (logistic regression analyses)

| Variables | Univariate analyses | |  | Multivariable analysis  (continuous mFI scores) ^a^ | | Multivariable analysis (dichotomized mFI measure) ^b^ |  |
| --- | --- | --- | --- | --- | --- | --- | --- |
|  | OR (95% CI) | *P* value |  | OR (95% CI) | *P* value | OR (95% CI) | *P* value |
| Age | 1.045 (1.013-1.077) | 0.006 |  | - | - | - | - |
| Body mass index |  |  |  |  |  |  |  |
| 18.5-23.9 kg m^-2^ | Reference |  |  | Reference |  | Reference |  |
| <18.5 kg m^-2^ | 3.076 (1.584 to 5.972) | 0.001 |  | 3.097 (1.532 to 6.260) | 0.002 | 2.823 (1.396 to 5.709) | 0.004 |
| ≥24 kg m^-2^ | 1.295 (0.845 to 1.983) | 0.235 |  | 1.343 (0.863 to 2.090) | 0.191 | 1.426 (0.918 to 2.216) | 0.115 |
| Continuous mFI scores | 1.345 (1.183 to 1.528) | <0.001 |  | 1.319 (1.151 to 1.511) | <0.001 | NA | NA |
| Frailty identified by mFI of ≥0.27 | 2.184 (1.440 to 3.313) | <0.001 |  | NA | NA | 2.059 (1.328 to 3.193) | 0.001 |
| Renal failure | 3.667 (0.958 to14.035) | 0.058 |  | - | - | - | - |
| Hypoalbuminemia |  |  |  |  |  |  |  |
| None | Reference |  |  |  |  | Reference |  |
| 30.0–39.9 g l^-1^ | 1.707 (1.118 to 2.607) | 0.013 |  | - | - | 1.346 (0.864 to 2.098) | 0.189 |
| <30.0 g l^-1^ | 3.921 (1.920 to 8.007) | <0.001 |  | - | - | 2.430 (1.138 to 5.187) | 0.022 |
| Na^+^ <135.0 mmol l^-1^ | 2.427 (1.373 to 4.291) | 0.002 |  | 1.865 (1.010 to 3.442) | 0.046 | - | - |
| Risk stratification of surgery by OSS ^c^ |  |  |  |  |  |  |  |
| High stress | Reference |  |  | Reference |  | Reference |  |
| Very high stress | 2.393 (1.354 to 4.227) | 0.003 |  | 1.973 (1.052 to 3.701) | 0.034 | 2.226 (1.227 to 4.038) | 0.008 |
| Duration of surgery (hour) | 1.181 (1.044 to 1.336) | 0.008 |  | - | - | - | - |

MFI, modified frailty index; NA, not applicable; Na+, serum natremia concentration; OSS, operative stress score.

^a^ Factors with *P* values <0.10 in univariate logistic regression analyses were included in the multivariable logistic regression model to identify the adjusted association between rising mFI score (per 0.09-point increase in mFI score) and the primary outcome. Except for frailty identified by mFI of ≥0.27, the variables in this table were all included in the multivariable model. The 11 components included in the mFI were excluded from the analyses. Multicollinearity was tested and not detected (all variance inflation factors <10). The multivariable logistic regression analysis was performed with the backward stepwise method. Hosmer-Lemeshow test for goodness of fit of the multivariable model: *χ*^2^=11.698, *df*= 8, *P=*0.165.

^b^ Factors with *P* values <0.10 in univariate logistic regression analyses were included in the multivariable logistic regression model to identify the adjusted association between dichotomized mFI measure (i.e., mFI of ≥0.27) and the primary outcome. Except for continuous mFI scores, the variables in this table were all included in the multivariable analysis. The 11 components included in the mFI were excluded from the analyses. Multicollinearity was tested and not detected (all variance inflation factors <10). The multivariable logistic regression analysis was performed with the backward stepwise method. Hosmer-Lemeshow test for goodness of fit of the multivariable model: *χ*^2^=20.953, *df*= 8, *P=*0.07.

^c^ Identified the risk stratification of surgery by physiologic stress, i.e., operative stress score (OSS). The surgical procedures in the study were those with OSS level 4 (i.e., high stress) and OSS level 5 (i.e., very high stress) [23]. Detailed data on surgery procedures are provided in Supplemental Table 1.

**Supplementary Table 7** Association of RAI-rev with postoperative life-threatening morbidity and mortality (logistic regression analyses)

| Variables | Univariate analyses | |  | Multivariable analysis  (continuous RAI-rev scores) ^a^ | | Multivariable analysis  (dichotomized RAI-rev measure) ^b^ | |
| --- | --- | --- | --- | --- | --- | --- | --- |
|  | OR (95% CI) | *P* value |  | OR (95% CI) | *P* value | OR (95% CI) | *P* value |
| Body mass index |  |  |  |  |  |  |  |
| 18.5-23.9 kg m^-2^ | Reference |  |  | Reference |  | Reference |  |
| <18.5 kg m^-2^ | 3.076 (1.584 to 5.972) | 0.001 |  | 2.976 (1.473 to 6.014) | 0.002 | 2.960 (1.474 to 5.943) | 0.002 |
| ≥24 kg m^-2^ | 1.295 (0.845 to 1.983) | 0.235 |  | 1.368 (0.879 to 2.128) | 0.165 | 1.317 (0.844 to 2.056) | 0.226 |
| Continuous RAI-rev scores | 1.066 (1.032 to 1.100) | <0.001 |  | 1.052 (1.018 to 1.087) | 0.002 | NA | NA |
| Frailty identified by RAI-rev of ≥45 | 2.199 (1.443 to 3.353) | <0.001 |  | NA | NA | 1.862 (1.188 to 2.919) | 0.007 |
| Hypertension | 1.409 (0.943 to 2.106) | 0.095 |  | - | - | - | - |
| Coronary heart disease | 2.087 (1.337 to 3.256) | 0.001 |  | 1.886 (1.176 to 3.026) | 0.008 | 1.900 (1.185 to 3.046) | 0.008 |
| Previous stroke | 1.847 (1.164 to 2.933) | 0.009 |  | - | - | - | - |
| Diabetes mellitus | 1.694 (1.120 to 2.562) | 0.012 |  | - | - | - | - |
| Hypoalbuminemia |  |  |  |  |  |  |  |
| None | Reference |  |  |  |  | Reference |  |
| 30.0–39.9 g l^-1^ | 1.707 (1.118 to 2.607) | 0.013 |  | - | - | 1.369 (0.878 to 2.134) | 0.166 |
| <30.0 g l^-1^ | 3.921 (1.920 to 8.007) | <0.001 |  | - | - | 2.341 (1.092 to 5.018) | 0.029 |
| Na^+^ <135.0 mmol l^-1^ | 2.427 (1.373 to 4.291) | 0.002 |  | - | - | - | - |
| Risk stratification of surgery by OSS ^c^ |  |  |  |  |  |  |  |
| High stress | Reference |  |  | Reference |  | Reference |  |
| Very high stress | 2.393 (1.354 to 4.227) | 0.003 |  | 2.369 (1.309 to 4.288) | 0.004 | 1.970 (1.052 to 3.689) | 0.034 |
| Duration of surgery (hour) | 1.181 (1.044 to 1.336) | 0.008 |  | - | - | - | - |

RAI-rev, Revised-Risk Analysis Index; NA, not applicable; Na+, serum natremia concentration; OSS, operative stress score.

^a^ Factors with *P* values <0.10 in univariate logistic regression analyses were included in the multivariable logistic regression model to identify the adjusted association between rising RAI-rev score (per 1-point increase in RAI-rev score) and the primary outcome. Except for frailty identified by RAI-rev of ≥45, the variables in this table were all included in the multivariable model. Age, sex, cancer, poor appetite, unintentional weight loss, renal failure, congestive heart failure, shortness of breath, living status, cognitive decline, and functional status were excluded because they were included in the RAI-rev. Multicollinearity was tested and not detected (all variance inflation factors <10). The multivariable logistic regression analysis was performed with the backward stepwise method. Hosmer-Lemeshow test for goodness of fit of the multivariable model: *χ*^2^=7.647, *df*= 8, *P=*0.469.

^b^ Factors with *P* values <0.10 in univariate logistic regression analyses were included in the multivariable logistic regression model to identify the adjusted association between dichotomized RAI-rev measure (i.e., RAI-rev of ≥45) and the primary outcome. Except for continuous RAI-rev scores, the variables in this table were all included in the multivariable model. Age, sex, cancer, poor appetite, unintentional weight loss, renal failure, congestive heart failure, shortness of breath, living status, cognitive decline, and functional status were excluded because they were included in the RAI-rev. Multicollinearity was tested and not detected (all variance inflation factors <10). The multivariable logistic regression analysis was performed with the backward stepwise method. Hosmer-Lemeshow test for goodness of fit of the multivariable model: *χ*^2^=8.065, *df*= 8, *P=*0.427.

^c^ Identified the risk stratification of surgery by physiologic stress, i.e., operative stress score (OSS). The surgical procedures in the study were those with OSS level 4 (i.e., high stress) and OSS level 5 (i.e., very high stress) [23]. Detailed data on surgery procedures are provided in Supplemental Table 1.

**Supplementary** **Table 8** Association of dichotomized mFI measure with life-threatening morbidity and mortality (survival analysis)

| Variables | Univariate analyses ^a^ | |  | Multivariable analysis ^b^ |  |
| --- | --- | --- | --- | --- | --- |
|  | Hazard Ratio (95% CI) | *P* value |  | Hazard Ratio (95% CI) | *P* value |
| Age | 1.038 (1.008 to 1.070) | 0.014 |  | 1.030 (0.998 to 1.062) | 0.066 |
| Body mass index |  |  |  |  |  |
| 18.5-23.9 kg m^-2^ | Reference |  |  | Reference |  |
| <18.5 kg m^-2^ | 2.610 (1.400 to 4.864) | 0.003 |  | 2.412 (1.253 to 4.640) | 0.008 |
| ≥24 kg m^-2^ | 1.329 (0.879 to 2.012) | 0.178 |  | 1.431 (0.936 to 2.188) | 0.098 |
| Frailty identified by mFI of ≥0.27 | 2.161 (1.456 to 3.206) | <0.001 |  | 2.042 (1.353 to 3.083) | 0.001 |
| Renal failure | 3.408 (1.080 to 10.75) | 0.036 |  | 3.611 (1.102 to 11.84) | 0.034 |
| Chronic hepatic dysfunction ^c^ | 1.848 (0.933 to 3.663) | 0.078 |  | 1.141 (0.526 to 2.476) | 0.738 |
| Hypoalbuminemia |  |  |  |  |  |
| None | Reference |  |  | Reference |  |
| 30.0–39.9 g l^-1^ | 1.539 (1.019 to 2.325) | 0.040 |  | 1.222 (0.796 to 1.875) | 0.360 |
| <30.0 g l^-1^ | 3.551 (1.869 to 6.746) | <0.001 |  | 2.135 (1.078 to 4.230) | 0.030 |
| Na^+^ <135.0 mmol l^-1^ | 2.183 (1.281 to 3.720) | 0.004 |  | 1.496 (0.845 to 2.651) | 0.167 |
| Risk stratification of surgery by OSS ^d^ |  |  |  |  |  |
| High stress | Reference |  |  | Reference |  |
| Very high stress | 2.415 (1.435 to 4.063) | 0.001 |  | 1.797 (0.997 to 3.240) | 0.051 |
| Duration of surgery (hour) | 1.170 (1.043 to 1.312) | 0.008 |  | 1.095 (0.968 to 1.239) | 0.147 |

MFI, modified frailty index; Na+, serum natremia concentration; OSS, operative stress score.

^a^ Survival analysis and log-rank test.

^b^ Multivariable Cox proportional hazards regression model adjusted for the factors with *P* values <0.10 screened by univariate survival analyses. The factors in this table were all included in the multivariable Cox regression model with enter method.

^c^ Refers to hepatic impairment classified as Child-Pugh class B and C.

^d^ Identified the risk stratification of surgery by physiologic stress, i.e., operative stress score (OSS). The surgical procedures in the study were those with OSS level 4 (i.e., high stress) and OSS level 5 (i.e., very high stress) [23]. Detailed data on surgery procedures are provided in Supplemental Table 1.

**Supplementary Table 9** Association of dichotomized RAI-rev measure with life-threatening morbidity and mortality (survival analysis)

| Variables | Univariate analyses ^a^ | |  | Multivariable analysis ^b^ | |
| --- | --- | --- | --- | --- | --- |
|  | Hazard Ratio (95% CI) | *P* value |  | Hazard Ratio (95% CI) | *P* value |
| Body mass index |  |  |  |  |  |
| 18.5-23.9 kg m^-2^ | Reference |  |  | Reference |  |
| <18.5 kg m^-2^ | 2.610 (1.400 to 4.864) | 0.003 |  | 2.416 (1.257 to 4.645) | 0.008 |
| ≥24 kg m^-2^ | 1.329 (0.879 to 2.012) | 0.178 |  | 1.325 (0.865 to 2.030) | 0.197 |
| Frailty identified by RAI-rev of ≥45 | 2.181 (1.464 to 3.248) | <0.001 |  | 1.822 (1.198 to 2.770) | 0.005 |
| Coronary heart disease | 2.146 (1.413 to 3.257) | <0.001 |  | 1.946 (1.254 to 3.019) | 0.003 |
| Previous stroke | 1.741 (1.122 to 2.701) | 0.013 |  | 1.495 (0.935 to 2.344) | 0.080 |
| Diabetes mellitus | 1.745 (1.176 to 2.589) | 0.006 |  | 1.551 (1.030 to 2.335) | 0.036 |
| Chronic hepatic dysfunction ^c^ | 1.848 (0.933 to 3.663) | 0.078 |  | 1.118 (0.523 to 2.394) | 0.773 |
| Hypoalbuminemia |  |  |  |  |  |
| None | Reference |  |  | Reference |  |
| 30.0–39.9 g l^-1^ | 1.539 (1.019 to 2.325) | 0.040 |  | 1.227 (0.801 to 1.879) | 0.346 |
| <30.0 g l^-1^ | 3.551 (1.869 to 6.746) | <0.001 |  | 2.113 (1.068 to 4.180) | 0.032 |
| Na^+^ <135.0 mmol l^-1^ | 2.183 (1.281 to 3.720) | 0.004 |  | 1.326 (0.746 to 2.357) | 0.336 |
| Risk stratification of surgery by OSS ^d^ |  |  |  |  |  |
| High stress | Reference |  |  | Reference |  |
| Very high stress | 2.415 (1.435 to 4.063) | 0.001 |  | 1.908 (1.057 to 3.446) | 0.032 |
| Duration of surgery (hour) | 1.170 (1.043 to 1.312) | 0.008 |  | 1.093 (0.964 to 1.239) | 0.164 |

RAI-rev, Revised-Risk Analysis Index; Na+, serum natremia concentration; OSS, operative stress score.

^a^ Survival analysis and log-rank test.

^b^ Multivariable Cox proportional hazards model adjusted for the factors with *P* values <0.10 screened by univariate survival analyses. The factors in this table were all included in the multivariable Cox regression model with enter method.

^c^ Refers to hepatic impairment classified as Child-Pugh class B and C.

^d^ Identified the risk stratification of surgery by physiologic stress, i.e., operative stress score (OSS). The surgical procedures in the study were those with OSS level 4 (i.e., high stress) and OSS level 5 (i.e., very high stress) [23]. Detailed data on surgery procedures are provided in Supplemental Table 1.

Supplementary Figure 1


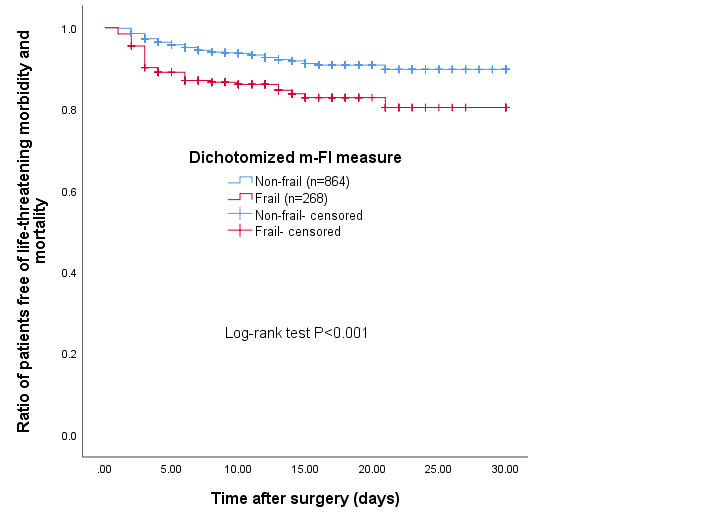


Supplementary Figure 1: The development of life-threatening morbidity and mortality between frail (identified by mFI of ≥0.27) and non-frail (identified by mFI of <0.27) patients. Compared with non-frail patients, the frail patients had a shortened time (mean time: 25.567 days [95%CI 24.301 to 26.833] vs. 27.826 days [95%CI 27.289 to 28.363]; log-rank test: *P*<0.001) to develop 30-day life-threatening morbidity and mortality. **M**FI, modified frailty index.

Supplementary Figure 2


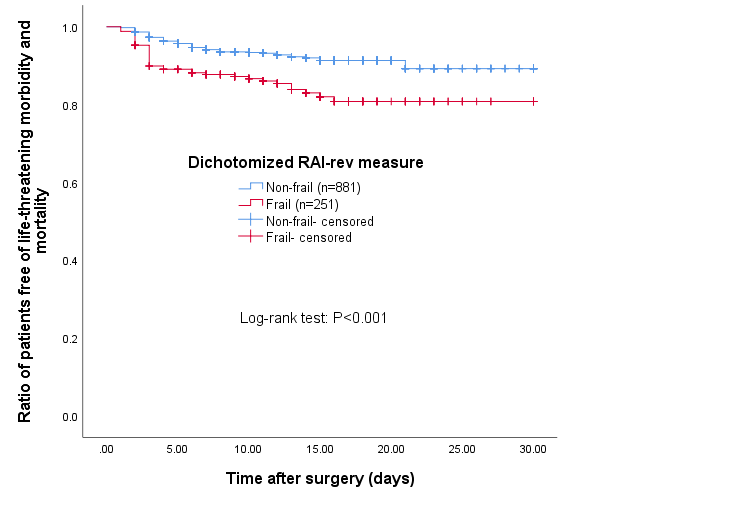


Supplementary Figure 2: The development of life-threatening morbidity and mortality between frail (identified by RAI-rev of ≥45) and non-frail (identified by RAI-rev of <45) patients. Compared with non-frail patients, the frail patients had a shortened time (mean time: 25.506 days [95%CI 24.209 to 26.802] vs. 27.780 days [95%CI 27.230 to 28.330]; log-rank test: *P*<0.001) to develop 30-day life-threatening morbidity and mortality. RAI-rev, revised Risk Analysis index.
